# Cardiac Fibroelastoma versus Thrombus: Echocardiographic Evidence Can Be Misleading

**DOI:** 10.1155/2016/2896056

**Published:** 2016-07-31

**Authors:** John P. O'Laughlin, Gautam Verma, Iosif Gulkarov

**Affiliations:** ^1^Department of Internal Medicine, New York Methodist Hospital, 506 6th Street, Brooklyn, NY 11215, USA; ^2^Department of Cardiothoracic Surgery, New York Methodist Hospital, 263 7th Avenue, Brooklyn, NY 11215, USA; ^3^Department of Cardiothoracic Surgery, Weill Cornell Medicine, 68th Street M404, New York, NY 10021, USA

## Abstract

We present a case of a young female with stroke symptoms who underwent valve sparing resection of a presumed fibroelastoma based on echocardiographic findings. After confirming embolic stroke, she underwent excision of the lesion, which on pathology revealed a nonbacterial thrombus. Ultimately, this led to a more extensive work-up leading to the discovery of a papillary serous ovarian carcinoma, the underlying cause of her hypercoagulable state. The initial echocardiographic findings painted the clear picture of a papillary tumor on the aortic valve which was likely the source of the emboli resulting in ischemic stroke. This unique case presentation illustrates that imaging, including echocardiography, may not always coincide with the clinical diagnosis. Thus, understanding the differential diagnoses of cardiac masses is of vital clinical significance. The distinction of fibroelastoma versus the much less common finding of aortic thrombus may lead to early diagnosis of malignancy and prevention of life threatening events due to stroke or undiagnosed disease.

## 1. Introduction

The incidence of primary cardiac tumors is rare with the top three most common tumors consisting of myxomas, lipomas, and fibroelastomas (in order of respective occurrence) [1–3]. Fibroelastomas are described as having a papillary structure and may be found throughout the endocardial tissues, with an affinity for valvular structures [4]. Clinical presentation of this tumor can vary with most patients presenting asymptomatic with incidental finding on echocardiography. Location of the tumor can impact clinical presentation. Tumors on the right side of the heart are commonly asymptomatic or patients may present with pulmonary emboli [5], whereas left sided tumors can result in life threatening embolic phenomena [5]. This is a result of the fragile nature of the tumor's composition and their high affinity for platelet aggregation [5].

Echocardiography remains the cornerstone for identification of cardiac tumors, but the diagnosis based on imaging is not always definitive. Cardiac masses have variations in shape and size making it difficult to diagnose a tumor versus a thrombus based on imaging findings and clinical presentation. The sensitivity and specificity have been estimated to be near 90% when using transthoracic echocardiography in diagnosis of papillary fibroelastoma if tumor size is greater than 2 mm [5]. However, there is still room for an alternative diagnosis. We present a unique case of a presumed aortic valve fibroelastoma that after valve sparing excision turned out to be a nonbacterial thrombus.

## 2. Case

**Figure 1:**
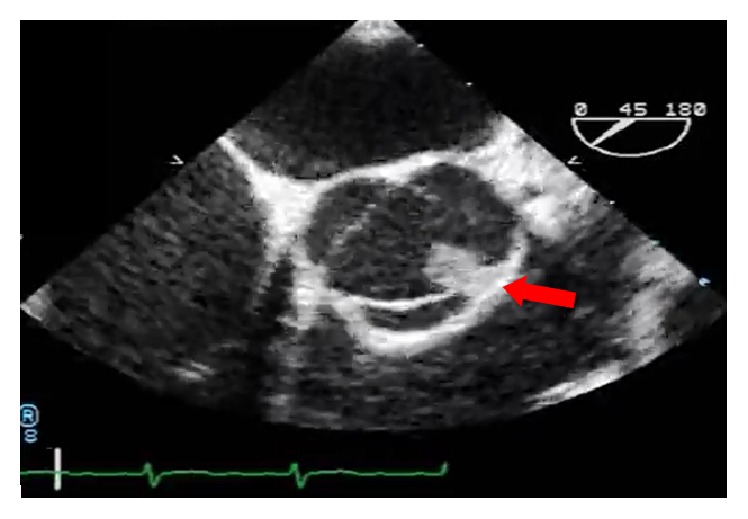
Transesophageal echocardiograph illustrating large, papillary, solid, fixed mass, measuring 7 × 7 mm on the left coronary cusp, on the left ventricular aspect of the aortic valve.

**Figure 2:**
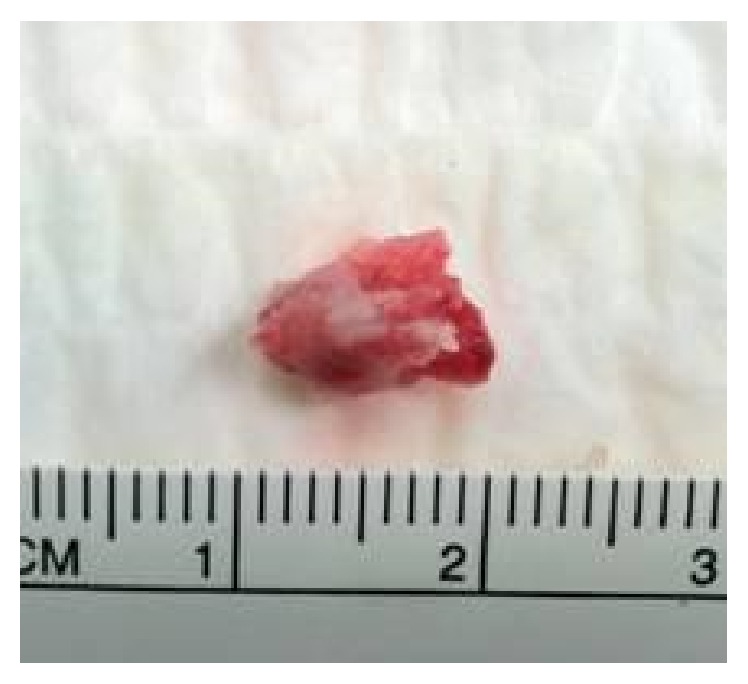
Mass surgically excised from left coronary cusp of aortic valve, approximately 9 × 5 mm. Pathology consistent with thrombus.

A 26-year-old previously healthy female presented to emergency department with complaints of headaches for two weeks associated with right sided numbness, weakness, diplopia, facial droop, dysarthria, and ataxia. The patient underwent work-up for a cerebral vascular accident. MRI confirmed the diagnosis of acute stroke involving the left posterior cerebral artery territory with smaller foci of ischemia in various cerebral territories, consistent with embolic phenomena. Transesophageal echocardiography revealed a large, papillary, solid, fixed mass, measuring 7 × 7 mm on the left coronary cusp (Figure 1) of the aortic valve that was suspected to be an aortic fibroelastoma due to its papillary structure and size. She underwent valve sparing excision of the fibroelastoma (Figure 2). Pathology later returned consistent with thrombus. The patient was recommended to have a hypercoagulable work-up as an outpatient but was lost to follow-up.

Four months after the initial surgery she presented with complaints of left lower quadrant abdominal pain, described as sharp and stabbing in nature. CT scan of the abdomen and pelvis revealed massive ascites. Recommended correlation study with transvaginal ultrasound was conducted and revealed a complex mass of mixed echogenicity with mildly increased vascularity measuring 7.6 × 7.2 × 9.0 cm, located posterior to the uterus and extending to the right adnexa. Biopsy of the lesion revealed adenocarcinoma. She went on to undergo bilateral salpingooophorectomy, omentectomy, lysis of adhesions, and peritoneal biopsies confirming a final diagnosis of papillary serous ovarian carcinoma, Stage IIc.

## 3. Discussion

Stroke-like symptoms in otherwise healthy young adults can be very alarming. Initial assessment involves multiple brain imaging modalities before a suspected diagnosis is made, as strokes can be ischemic or hemorrhagic in nature. Cardiac emboli are a common source of ischemic stroke and may be the result of bacterial or nonbacterial thrombi, or cardiac tumors. Due to the vast differential of cardiac masses, assessment and diagnosis pose a challenging task for physicians, especially cardiac tumors. Nonneoplastic processes such as thrombus and valvular vegetations can mimic tumors; therefore physicians should consider them as part of the differential diagnosis.

Cardiac tumors can be categorized into primary cardiac neoplasms or metastatic deposits from other remote primary neoplasms; most commonly breast, lung, lymphomas, melanomas, and renal tumors [6]. Primary cardiac neoplasms can be either benign or malignant. The most common malignant tumors include angiosarcoma and rhabdomyosarcoma. Benign lesions include myxomas, lipomas, and fibroelastomas and represent the most frequent type of intracardiac lesions [1–3]. Myxomas are the most common benign primary cardiac tumor seen in patients 30–60 years of age with increased incidence in women [7]. Myxomas are solitary tumors which arise from the interatrial septum normally within the left atrium [8, 9]. Lipomas are the second most common tumor, occurring in middle to late adulthood, composed of adipocytes, and occur in all chambers within the heart [10, 11]. Fibroelastomas are the third most common tumor, also presenting in middle to late adulthood [11]. They are commonly described as having a papillary structure and can arise from any endocardial surface, but most are found on the aortic or mitral valves [4, 11].

The occurrence of primary cardiac neoplasms is rare and diagnosed in about 0.001–0.03% of echocardiographic studies, reported by Sutsch et al., 1991, based an analysis of 20,305 echocardiographs [12]. Transthoracic or transesophageal echocardiography is the primary imaging modality in initial work-up of cardiac tumors. Transthoracic echocardiography (TTE) is a convenient imaging modality as it is cost effective, noninvasive, and provides efficient results. Within minutes clinicians have vital information regarding the shape, size, mobility, and precise location of masses within the heart [13]. Transesophageal echocardiography (TEE), though similar to TTE, provides clinicians with superior imaging when compared to TTE due to better acoustic windows resulting from lack of signal loss from penetration through adjacent thoracic wall and lung structures [14]. As a result, better visualization helps to identify morphology of smaller cardiac tumors, less than 5 mm, that may otherwise be missed on TTE [14]. TEE is more invasive and requires mild anesthesia. TEE is usually only warranted if there is a high clinical suspicion for a cardiac mass not seen on TTE or lack of identifiable characteristics of a mass on TTE due to poor acoustic windows [6]. Enhancement of masses during echocardiography with the use of a contrast medium may help to further elucidate whether a cardiac mass is a tumor or thrombus [6, 15]. However, this is only beneficial if the tumor is of malignant origin as these tumors are highly vascularized and will enhance on imaging [6, 15, 16]. The use of contrast echocardiography in distinguishing between benign cardiac tumors and thrombi provides no clinical benefit and is unreliable due to their lack of vascularity and enhancement [6, 16].

Treatment of cardiac masses normally depends on the clinical presentation and type of tumor. There is no defined protocol nor evidence based approach to treating these patients; thus clinical judgement in coordination with imagery must be utilized. Benign tumors, including papillary fibroelastomas, are commonly surgically excised due to the high risk of embolic phenomena [17]. On the other hand, malignant tumors typically have poor outcomes despite surgical intervention due to metastatic disease and high rates of recurrence [6, 18].

Our patient presented with stroke due to thromboembolic disease from aortic valve vegetation that was believed to be a papillary fibroelastoma due to its papillary structure on echocardiography and patient's lack of clinical evidence of bacterial endocarditis. TTE and TEE studies revealed findings consistent with a large papillary lesion. Due to the acute risk of further embolic phenomena valve sparing excision was performed revealing a nonbacterial thrombus. Even with the use of contrast echocardiography or cardiac MRI, we would not have been able to differentiate between thrombus and papillary fibroelastoma [19]. Therefore, the benefits of pursuing excision outweighed the risk of surgery as the patient was at high risk for potentially fatal stroke events.

## 4. Conclusion

Echocardiographic imaging can only provide limited information on the characteristic morphology of cardiac masses, with further contribution to pathologic etiology improved with use of contrast echocardiography or cardiac MRI (limited availability at most institutions). Despite clinical objective evidence supporting benign cardiac tumor etiology (i.e., TEE study consistent with papillary fibroelastoma), one must take the entire clinical picture into account before pursuing invasive surgical procedures. This unique case provides an example that medicine and surgery is not always “textbook” in nature. Even though the echocardiographic evidence supported our clinical suspicion, pathologic analysis did not coincide. Further investigative research must be done to help clinicians and surgeons diagnostically differentiate benign cardiac tumors (myxomas, papillary fibroelastomas, and lipomas) from the less commonly occurring native aortic thrombus. We also recommend that when aortic thrombi are suspected or discovered on imaging, an age-appropriate malignancy work-up should be performed as approximately 15% of cancer patients may have thromboembolic events throughout their disease process [20].

## Abbreviations

TTE: Transthoracic echocardiograph
TEE: Transesophageal echocardiograph
MRI: Magnetic Resonance Imaging.
